# Systematic Review of Current Audiological Treatment Options for Patients with Treacher Collins Syndrome (TCS) and Surgical and Audiological Experiences of an Otorhinolaryngologist with TCS

**DOI:** 10.3390/jpm14010081

**Published:** 2024-01-10

**Authors:** Ivana Marinac, Robert Trotić, Andro Košec

**Affiliations:** 1 Department of Otorhinolaryngology, Čakovec County Hospital, 40000 Čakovec, Croatia; dr.ivana.marinac@gmail.com; 2 Department of Otorhinolaryngology and Head and Neck Surgery, University Hospital Center Sestre Milosrdnice, 10000 Zagreb, Croatia; trotic@gmail.com; 3 School of Dental Medicine, University of Zagreb, 10000 Zagreb, Croatia; 4 School of Medicine, University of Zagreb, 10000 Zagreb, Croatia

**Keywords:** Treacher Collins syndrome, conductive hearing loss, rehabilitation

## Abstract

Treacher Collins syndrome (TCS) is a rare congenital craniofacial condition that affects approximately one out of fifty thousand births. Different ratios of TCS patients have conductive hearing loss: 88%^1^ vs. 91.4–100.00%^2^. For this reason, it was examined which hearing solutions can be used with this condition and how effective they are. A systematic literature review was conducted, which showed that the bone-anchored hearing aid (BAHA, OSIA), the bone conduction implant (Bonebridge) or the active implant of the middle ear (Soundbridge) are reliable methods for the treatment of conductive hearing loss in TCS patients. After the implantation of all available hearing solutions, improved hearing and speech comprehension were observed. Additionally, a statement regarding the treatment of TCS and a personalized point of view of a clinical expert with TCS were provided. However, due to the small amount of data, no general recommendations can be given for the treatment of hearing loss in TCS patients; therefore, it is advised to collect more data on hearing solutions for TCS patients in future research.

## 1. Introduction

Treacher Collins syndrome (TCS) (OMIM 154500, 613717, 248390, 618939) is a rare condition with an incidence of 1:50,000 live births [1]. The British surgeon and distinguished ophthalmologist, Sir Edward Treacher Collins, is given credit for first describing the syndrome in 1900 and giving it its name [2]. There are two earlier reports by Thomson in 1846 and Berry in 1889 [1]. Other given names of this condition were Franceschetti–Zwahlen–Klein syndrome, suggested by Franceschetti and Klein later in 1949, and mandibulofacial dysostosis.

The gene responsible for most cases of TCS is TCOF1, which was shown to be expressed in the embryonic neural folds and in the developing branchial or pharyngeal arches at critical stages of morphogenesis. Mutations within TCOF1 cause defects in the structure and function of Treacle resulting in the protein that is noxious to craniofacial development. Mutated Treacle and RNA polymerase activity leads to a significant deficiency of neural crest cells for complete formation of the branchial arch 1 and 2 derivatives for the craniofacial bones, cartilages and soft tissues. While mutations in TCOF1 are responsible for most cases of TCS, there are three other genes more recently implicated in rarer forms of the syndrome, POLR1B, POLR1C and POLR1D. Mutations in these three genes also lead to craniofacial cartilage hypoplasia and osseous malformations characteristic of TCS [1,2,3].

The condition TCS is a rare congenital craniofacial condition which gets distinguished by a number of developmental anomalies that are related to the head and neck, such as hypoplasia of the zygomatico-maxillary complex, colobomas of the lower eyelids, downward slanting of the palpebral fissures, cleft palate, mandibular hypoplasia, micrognathia and retrognathia, airway dysfunction and malformation of the outer and middle ear [1,2,3,4,5,6,7]. Typically, there are also bilateral and symmetric abnormalities of the auricle (microtia) and middle ear malformations that can cause conductive hearing loss (CHL) [1,2,3,5,8]. A great proportion of TCS patients have bilateral CHL: 88%^1^ vs. 91.4–100.00% [1,2]. Microtia and atresia of the external auditory canal, as well as malformations of the middle ear ossicles, are a few examples of possible conditions regarding the auditory pathway, but the inner ear remains normal and healthy in most TCS patients [5]. In general, the treatment options for CHL range from conventional hearing aids and passive percutaneous or active transcutaneous bone conduction devices to active middle ear implants [9,10]. The first ever used were conventional hearing aids, which amplify the sounds captured so that they can be transmitted to the middle and inner ear if an auricle or ear canal is developed so that it can safely wear it or it is mounted on a headband. The bone-anchored hearing aid system from the company Cochlear (Cochlear Limited, Sydney, Australia) or BAHA/OSIA is available in non-invasive, percutaneous, and transcutaneous versions. With the non-invasive BAHA softband system, the audio processor is put in the mastoid area with a headband in order to be able to transmit the sounds [11]. In the percutaneous passive bone-anchored hearing aid, an abutment screwed in the temporal bone protrudes through the skin, and the audio processor is attached to it. For the transcutaneous model, the implant is embedded in the bone under the skin and the signals from the audio processor are transmitted passively through the skin [12]. However, there are also active transcutaneous bone-anchored hearing aids, such as the Bonebridge from the company MED-EL (MED-EL, Innsbruck, Austria), which transmits the vibrations of the sounds directly to the skull bone, thus enabling hearing [13]. MED-EL also offers a non-invasive bone conduction hearing aid that can be adhesively attached behind the ear, the ADHEAR. In addition, there are active middle ear implants from MED-EL. An example of the latter is the Vibrant Soundbridge (VSB), which can replace active structures in the middle ear and at the same time stimulate the other components to transmit sound waves to the inner ear [14,15].

It is sometimes difficult to make a definitive diagnosis of TCS based on clinical appearance only because the number and intensity of signs and symptoms of the syndrome vary greatly, from subtle to severe, and there are clinical overlaps between TCS, Goldenhar, Nager and Miller syndromes, all resulting from alterations of the development of the first and second pharyngeal arches [7,8]. Although there is much resemblance between these syndromes, the definitive diagnosis should be based on genetic testing to promote further research of gene mutations. Considering the molecular analysis of TCS patients, the inheritance pattern is mostly autosomal dominant, but there are reported autosomal recessive cases of the condition, based on mutations of the TCOF1, POLR1C, POLR1D, as well as POLR1B—source: OMIM database [1,5]. It is reported that about 60% of TCS patients present as “de novo” mutations [1].

One particularly important aspect of the condition is the psychological and sociological effects achieved through a good psychosocial adjustment, experiencing an increasing acceptance of self and social acceptance over time, and demonstrated adaptive strategies like optimism, motivation and positive attitude [16,17].

A lot of individuals with TCS have to deal with sleep apnea and comorbidities that make breathing difficult, which makes surgical procedures complicated because ventilation and intubation are typically challenging [18]. As there is a wide range of symptoms for this disease, a multidisciplinary team is usually required to effectively address the various pathologies [3]. The disciplines range from the ear, nose and throat department, orthodontics, geneticists, audiologists and pediatricians to craniofacial plastic surgeons [3,7].

Due to the variety of conditions and approaches regarding the treatment of TCS, only a limited number of publications deal with the hearing loss (HL) conditions of TCS patients. Therefore, the aim of this systematic review was to identify the state-of-the-art approach for providing a suitable hearing solution for TCS patients with CHL and extract the outcomes of the performed audiological assessments after a hearing loss treatment and/or rehabilitation. To review suitable publications, the following PICOS were applied for the search in the PubMed database: Population—Individuals of any gender or ethnicity with Treacher Collins syndrome (TCS). Intervention—Hearing loss treatment (any kind of hearing device). Comparators—not applicable. Outcomes—Data regarding audiological outcomes, language acquisition, hearing ability, general performance, quality of life, satisfaction and subjective outcomes. Study design—All study designs were included. Systematic reviews, letters, editorials and comments were not taken into consideration. Furthermore, experimental studies like animal or cadaver investigations were not included.

## 2. Materials and Methods

The systematic review was conducted and assembled according to the PRISMA guidelines (Supplementary file 1) [19]. The review is registered in the PROSPERO database, registration number 489604. In order not to exclude data prematurely, only the medical condition was searched for in the PubMed database. For this purpose, all possible synonyms for Treacher Collins syndrome were included for the search terms, which can be seen in Table 1 at the first search step. As a large number of unsuitable publications were displayed in the first search due to the TCS abbreviation, this term was removed from the search, so that 2211 publications were identified. The human filter was applied afterwards, and the search was specified for publications in the German and English languages. In the fourth search step in Table 1, a time limit of 10 years was set so that the most current treatment options would be provided. From the search date on 20 June 2023 at 13:00, 708 relevant articles were identified in the PubMed database in the last 10 years, which were considered for the further screening process.

The identified publications subsequently had to be screened in two stages, where the existing literature was examined for its usability for the systematic review according to the previously mentioned PICOS. The PICOS are shown in Table 2 which represent the inclusion criteria for possible appropriate papers. The exclusion criteria for the publications are also presented in Table 2.

**Figure 1:**
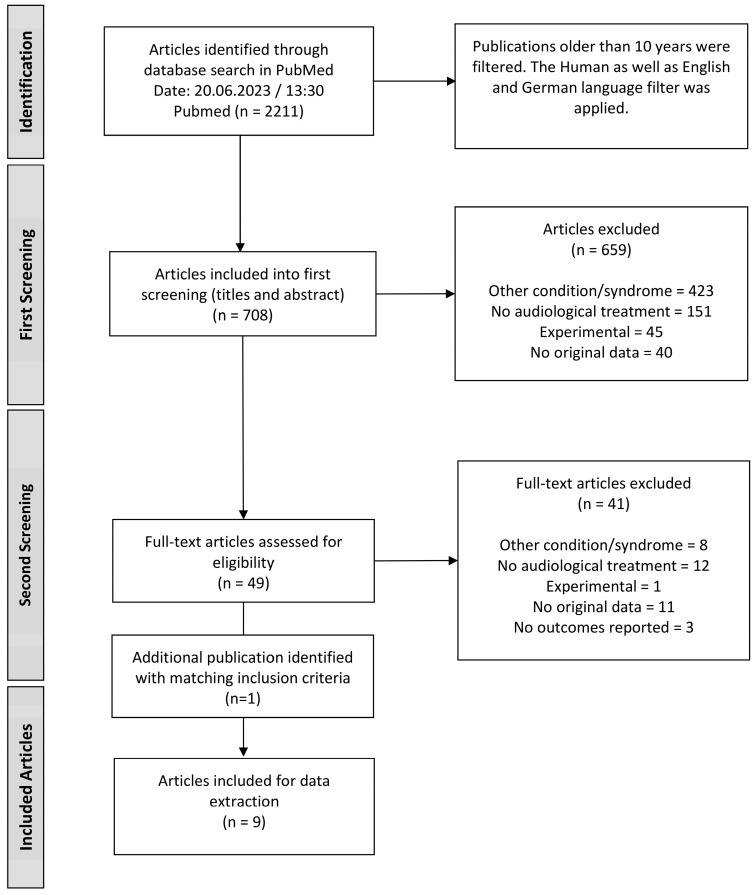
Flow chart diagram of the screening process according to PRISMA guidelines [19].

The screening process is described graphically in Figure 1. In the first stage of screening, the titles and abstracts were considered, and 659 publications were excluded using the criteria in Table 2. As shown in Figure 1, most of the literature had to be rejected because it dealt with other conditions, such as Goldenhar syndrome or Nager syndrome. In the second screening phase, the complete articles were examined, and 41 of the 49 publications had to be excluded. This left a total of eight publications that were eligible for data extraction. In the process of the literature search, an additional paper [20] was found that met the criteria, as it deals with Treacher Collins patients, describes audiological rehabilitation, and was also published in the last 10 years. Unfortunately, this publication was not included in the original search for unknown reasons but was added for the data collection of this systematic review due to the matching inclusion criteria. For this reason, data were collected from a total of nine publications.

The included papers were assessed using the Oxford Level of Evidence Guideline https://www.cebm.ox.ac.uk/resources/levels-of-evidence/ocebm-levels-of-evidence, (accessed on 15 November 2023). The publications can be divided into five categories, with level I denoting the highest conceivable level and level V denoting the lowest level. By looking at the study design, participant count or follow-up exams, for instance, the analysis shows how meaningful the study is. In general, larger participant populations and more follow-ups result in more expressive outcomes. Systematic reviews are often designated as level I or level II since they are generally superior to individual investigations. Case reports that present particular cases are typically level V. Additionally, it was recorded if the articles received funding or if there was a conflict of interest due to the possible bias of the results.

With pure tone audiometry, hearing ability can be tested. This is performed with a sound field measurement to determine the hearing threshold of the air conduction, in order to know at which frequency and volume the pure tones get perceived [21]. The pure tone average (PTA) includes four frequencies (0.5, 1, 2 and 4 kHz) and thus forms a mean value for the hearing threshold of a participant [22]. Since it is not only important to perceive tones but also to understand speech, speech audiometry also gets assessed. Here, words or sentences get presented at a certain decibel level, which then must be repeated by the test person. In this way, it can be recognized how well words can be understood by the participant. Understanding speech in turn offers the possibility of being able to participate actively in daily life without barriers [23].

In addition to the systematic literature review, an expert on the topic was interviewed to gain insight into the difficulties that need to be addressed with TCS and how a good hearing solution can have a positive impact on quality of life. Therefore, the otorhinolaryngologist Dr. Ivana Marinac from Zagreb, Croatia, who has Treacher Collins syndrome herself, provided an insightful perspective of the great wonder condition.

## 3. Results

The results are divided into an overview of the study cohort of the included studies, followed by a presentation of the outcomes of the audiological measurements performed in the publications and a review of the extracted literature. At the end, there is a personal, insightful perspective from the otorhinolaryngologist, Dr. Ivana Marinac, who herself has Treacher Collins syndrome.

### 3.1. Study Cohort Overview

The overview of the study cohort of included publications is presented in Table 3. In general, populations with Treacher Collins syndrome were included, but one study by Der et al. [24] provided a mixed study cohort where only three TCS patients were present in the whole study cohort of twenty-four people. Nevertheless, the data were taken into consideration, due to the promising outcomes for the audiological assessments.

As mentioned in the Introduction, the studies showed cohorts with typical TCS conditions. Most participants had jawbone hypoplasia, and in the case report of Massi et al. [25], the TCS patient also had a cleft palate. In terms of the hearing organ, most participants had a bilateral absence of the auricle. In the studies by Shih et al. [26] and Zhang et al. [27], atresia of the external auditory canal was also noted in the participants. In all studies from which data were extracted [14,20,22,24,25,26,27,28,29], bilateral craniofacial malformations were recognized in the subjects. As mentioned in most studies, the pathology of TCS occurs mostly due to a mutation of the nuclear protein TCOF1 gene, but there are other less frequent genes like POLR1A, POLR1B, POLR1C and POLR1D. Since the focus of the studies by Teber et al. [1], Vincent et al. [2], Fan et al. [22] and Zhang et al. [27] lies on gene sequencing, these studies stated that different variants of the TCOF1, and also other gene mutations, were found in the subjects. No statement was given in the remaining studies.

The individuals in the case reports of Massi et al. [25] and Sargsyan et al. [14] had mixed HL, which combines conductive and sensorineural HL conditions. In all other studies shown in Table 3, CHL was found in the TCS patients before treatment. Since the study by Asten et al. [28] was a mixed study group, people without HL or with sensorineural HL were also present.

Table 3 also shows which hearing solutions were applied to counteract the HL. It shows that conventional hearing aids were only applied in the study by Asten et al. [28]. It is important to note that in the mixed study group different hearing impairments were present. Otherwise, bone conduction devices were chosen because of the conductive hearing loss. Only in the study by Fan et al. [22], a bone-anchored hearing aid Ponto (Oticon, Ponto, Smørum, Denmark) was used, which is similar to the BAHA system, where a screw is fixated in the skull bone and brought out through the skin in order to place the audio processor there. Otherwise, the BAHA system and the Bonebridge were applied in most cases. As noted in Table 3, the studies by Massi et al. [25] and Zhang et al. [27] did not specify which device was implemented in particular, but only the general term bone conduction hearing solution or bone-anchored hearing aid with softband was given. The only active middle ear implant, the Vibrant Soundbridge (VSB), was mentioned in the case report from Sargsyan et al. [14]. In this publication, the proband first received a BAHA softband, which was then replaced by a VSB at the request of the parents and the child. The implantation took place at the age of six years. Similarly, the two children in the study by Sikolova et al. [20] were initially fitted with a BAHA softband system, which improved their ability to hear, but was then replaced with a unilateral Bonebridge in both participants because the wearing comfort of the BAHA softband system was not satisfactory. In the study by Rosa et al. [29], the mean age of implantation was nine years in four participants who received a percutaneous BAHA system. Prior to this, all participants wore conventional hearing aids.

Based on the overview of the applied hearing solutions, it can be concluded that bone conduction devices are still state-of-the-art for the treatment of conductive hearing loss in Treacher Collins patients. The BAHA system in various forms was the most utilized, as well as the Bonebridge.

**Table 3:** Overview of the included publications and the demographic data of the participants as well as an outline of the applied hearing devices.

Studies	* n * Subjects	Male	Female	Age Average [Years]	Age Min [Years]	Age Max [Years]	Hearing Loss (HL)	Used Devices
Asten et al., 2014 [28]	19	6	13	34	5	74	11× conductive HL 5× mixed HL 2× SNHL 1× normal hearing	6× BAHA bilateral 2× BAHA unilateral 5× behind-the-ear aids unilateral 2× behind-the-ear aids bilateral 1× in-the-ear hearing aids 3× no hearing device
Der et al., 2018 [24]	24 (3× TCS)	11	13	12	6	16	conductive HL	Bonebridge BC1601
Fan et al., 2019 [22]	13	4	9	-	-	-	conductive HL	1× softband BAHA 4× Ponto 1× Bonebridge BC1601
Massi et al., 2016 [25]	1	1	1	2	.	-	moderately severe mixed HL	bone-anchored hearing aid with softband (no company or model mentioned)
Rosa et al., 2016 [29]	9	2	7	1,6	0,1	14	bilateral conductive HL	4× BAHA
Sargsyan et al., 2014 [14]	1	-	1	6	-	-	congenital mixed HL	softband BAHA and Vibrant Soundbridge (VSB)
Shih et al., 2020 [26]	3 (only one TCS patient, only these data considered)	1	-	11	-	-	moderate to moderately severe conductive HL	BAHA BI300
Sikolova et al., 2023 [20]	2	1	1	6 and 7	-	-	bilateral conductive HL	previous BAHA softband, afterwards Bonebridge (BCI1602)
Zhang et al., 2013 [27]	7 (only data for one patient)	-	1	-		-	conductive HL	bone conduction hearing solution (no statement which device used)

### 3.2. Audiological Outcomes after Hearing Solutions

Although assessments such as generic quality of life questionnaires or subjective performance or satisfaction would have been considered in this review, these parameters were not found in any of the screened studies of the systematic review in combination with the use of hearing devices in TCS patients. Therefore, only the results of the audiological evaluations of the extracted studies are reported in Table 4. Almost all studies provide results of pure tone audiometry as well as speech audiometry.

In all studies except the one by Asten et al. [28], the air conduction hearing threshold was observed with the pure tone average (PTA) before surgery, which ranged between 55 dB and 70 dB, which is classified as a moderately severe hearing loss by the American Speech-Language-Hearing Association. In the study by Asten et al. [28], only the PTA values after the intervention with different hearing solutions were reported. For eleven measured participants, the PTA was about 52 dB, but it was not stated with which hearing devices these eleven people were fitted and what hearing loss was present before. Another noticeable aspect which can be seen in Table 4 is that in the case report by Sargsyan et al. [14] the pre-op and post-op thresholds are the same because the audio processor of the Vibrant Soundbridge was not activated for the sound field measurement. However, this also shows that there was no deterioration of the natural hearing. In all other studies, it was evident that both conventional hearing aids and the various bone conduction devices improved auditory capacity by an average of about 30 dB. It should be noted that in the study by Fan et al. [22], similar values were reported in the follow-up survey, but in this case, the improvement values were taken into consideration and not the values of the actual measurement after the operation. For this reason, these values are marked with a blue font in Table 4. The study by Shih et al. [26] provided no PTA values after the application of the hearing solution but reported that the patient consistently wore the device and experienced improved and satisfactory functionality.

Speech audiometry surveys were conducted in the publications by Asten et al. [28], Der et al. [24], Fan et al. [22], Massi et al. [25] and Sargsyan et al. [14]. Their results are presented in Table 4. Although different speech tests were administered, all results show a noticeable improvement in speech comprehension. In the study by Fan et al. [22], only the improvement values are given and not the actual percentage of speech intelligibility, which is why these values are also presented with a blue font in Table 4. The subjects in the other studies who conducted speech tests mostly achieved more than 90% speech understanding with the applied hearing solution. Only in the study by Asten et al. [28], the adolescents showed a slightly weaker performance with 77% overall, although 77% speech comprehension is a satisfactory outcome. The Speech, Spatial and Qualities of Hearing questionnaire (SSQ) is not a speech recognition assessment. It assesses the quality of life in relation to hearing impairment by checking how well the respondent understands speech and is able to hear spatially. In general, the participant indicates how satisfied they are with their general hearing ability. The scale for the SSQ questionnaire ranges from 0 to 10, with 10 being the best possible health-related quality of life. Table 4 shows that the quality of life of both TCS patients improved considerably 3 months after the Bonebridge implantation.

### 3.3. Paper Review

**Figure 2:**
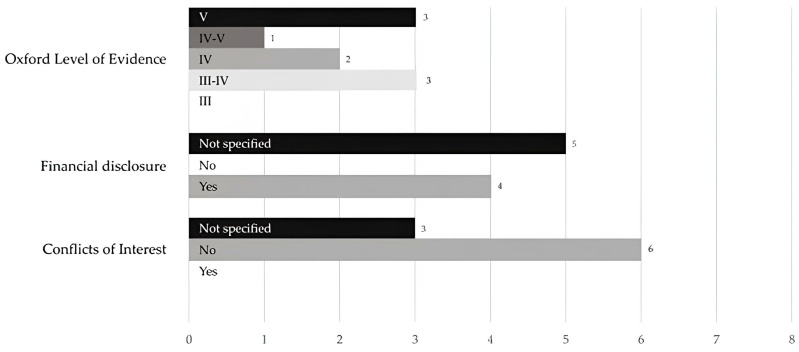
Systematic reviews included papers’ quality evaluation using the Oxford Level of Evidence, additional disclosure of financial assistance and potential conflicts of interest.

The publications mostly provided only a small number of study participants, which was noted in Table 3. Sometimes, only individual cases were presented. This is one of the reasons why the publications were not rated so well in the Oxford Level of Evidence evaluation. Due to the small and sometimes mixed study cohorts, as well as the small number of follow-up data acquisitions, the meaningfulness of the publications is sometimes not that high. Of the nine studies extracted, three publications were classified for the lowest level, which can also be seen in Figure 2. The highest level reached by three studies was the intermediate level III-IV. Additionally, four of the nine publications examined were financially supported, which can be seen in Figure 2. Most of the support came from scientific foundations or national research programs. Thus, no company was involved in the financial support. As shown in Figure 2, six publications made it clear that there was no conflict of interest. In the remaining studies, it was not stated whether a conflict of interest existed.

### 3.4. Perspective of Ivana Marinac, a Patient with TCS and an Otorhinolaryngologist

Part of my identity, a burden of my reality which shaped it, and will stay an integrative part of me—is conductive hearing loss. My CHL is an integrative part, among the other well-known characteristic signs of Treacher Collins syndrome. Many colleagues assume—all in all, that having CHL is just: Turn the radio up a bit! However, it is not. It is much more. Conductive hearing loss is all about speech intelligibility because although you hear words, there is a great challenge of dissociating them from background noise. You get a porridge instead of a symphony orchestra where you cannot pinpoint separately the instrument of your interest. Same as a simple conversation where all voices become the unrecognizable sound of—silence. Suddenly, being surrounded with many people but left in a silence because you cannot understand a word. In audiology, we call it the cocktail-party effect—especially hard to overcome for the hard-of-hearing and leading to social isolation and withdrawal. Although curious by nature, I could participate only in certain activities: conversations at a safe distance; needing enough time to understand what I had been told at the end separated me from people by giving me a burden of having possible intellectual difficulties instead of simple speech intelligibility issues.

After completing medical school, my residency choice came to me naturally. Who could better understand the reality of patients than a doctor who has the kind of hearing loss they have? A real environment of surgical masks, a lot of device noises in the operating theaters, and lousy room acoustics represented insurmountable difficulties.

I could not find myself in a new role. But there came a challenge—I was offered an opportunity—to participate in a study of a new bone conduction implant on the otologic horizon—the Bonebridge. There were many uncertainties regarding the efficacy of the new BCI in a Treacher Collins individual. My surgical situation was a shallow temporal bone and lowered sino-dural angle. My surgeon and I mutually decided to put the implant directly on the dura without knowing anything about how vibrations would act on brain tissue. There were no lifts in those first days. The curvature of my skull is different than usual, so it was necessary to fix the implant with a maxillofacial plate. Finally, we still did not know how it would work in these circumstances—the acoustic of a digital implant in a reality of an individual accustomed to an analog hearing aid. With the help of professional support and the persistence of my clinical engineers who took the challenge of fitting during the long and uncertain process of customizations. After some time, it became clear that I needed a second implant—the main challenge was how to handle an interaural transmission of signal, or will I be able to recognize the side of the sound input? Today, my bilaterality comes to me naturally. I have the ability of sound location, enriched sound and the opportunity to understand others in a loud environment: to fully participate in all activities around me. Even before I was introduced to Bonebridge, I truly believed that a bone-anchored world would eventually go “underground”, resolving the issue of skin complications. That led me through the process of making the decision about which implant to choose, and I am now implanted with bilateral Bonebridge implants—first generation BCI1601.

**Figure 3:**
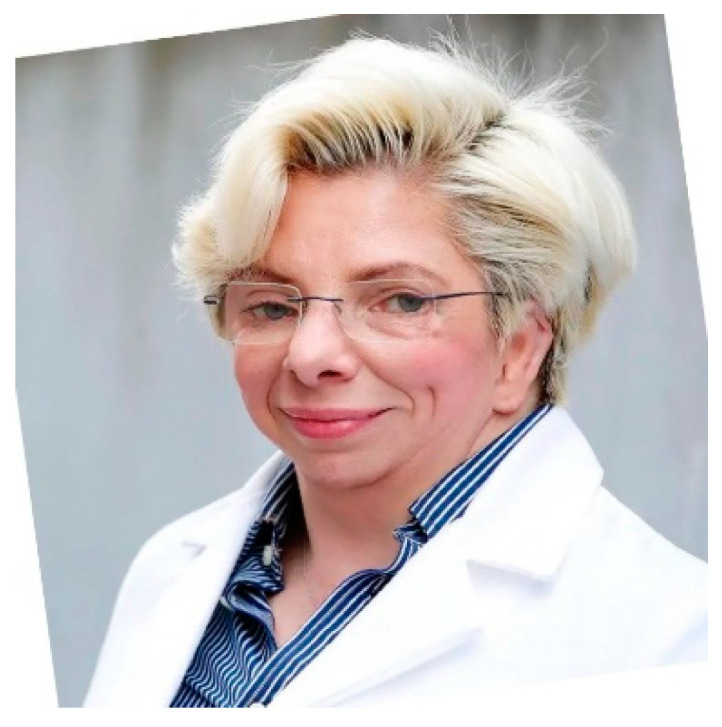
Dr. Ivana Marinac, an individual with TCS, an otorhinolaryngologist and a bilateral Bonebridge implant user.

There is a particularly important aspect of being born and living with TCS, but if it is taken wisely—a good psychosocial adjustment, although challenging, is not impossible (Figure 3). Achieving a professional career can help find a sense of purpose and fulfillment while building a strong support network. It allows, especially individuals with TCS, to showcase their skills and talents, proving that they are more than their physical appearance. Life-challenging experiences boost self-esteem and confidence, helping individuals with TCS to overcome any social challenge they may face. With determination and perseverance, demonstrated adaptive strategies like optimism, motivation and positive attitude can lead to a positive psychosocial adjustment for those living with this condition and excellent social acceptance over time.

In the end, I truly understand the saying—to stand on the shoulders of giants. Getting opinions and support, making decisions in a mutual process, many of my doctors became friends and helped me with creating an incredible life experience.

## 4. Discussion

It has been noticed in the screening process that there are some different syndromes that show similar symptoms and signs and are often characterized by resembling facial malformations. For this reason, the genetic mutations must also be taken into account in order to be able to differentiate which particular condition is present [27]. It was always necessary to pay close attention to whether the study examined did describe a differential diagnosis of TCS. Many publications have described Goldenhar or Nager syndrome or hemifacial microsomia, which is defined as a differential diagnosis of TCS [30]. Even if TCS patients were present, several studies had to be excluded from the review. Given the wide variety of manifestations and symptom types, many papers addressed airway management during surgery or facial reconstruction techniques in TCS patients but not hearing rehabilitation. Hearing loss and its treatment have therefore rarely been addressed and should be more focused on in future research.

In the results shown in Table 3, it can be seen that the participating TCS patients were quite young, mostly under 18. Only in the study group of Asten et al. [28] this was not the case, probably due to the mixed study group. Presumably, the participants were mainly under 18 because TCS is a congenital disease and therefore it is necessary to intervene as early as possible in order to counteract associated impairments. However, this could be a possible bias, as results may vary among children or adolescents because some audiological tests are more adult-oriented. This is a potential reason why individual speech tests were chosen for speech comprehension in order to best suit children and adolescents. In general, it was found that speech comprehension improved after the use of bone conduction devices in Treacher Collins patients, which is important because understanding speech and words can be an important factor for being able to participate in social life [23]. The speech audiometry results reported in the included studies are unfortunately not comparable with each other because different and partly individual assessments were chosen. Probably a variety of assessments was chosen in order to remain as flexible as possible so that adaptation to the individual conditions of the patients would be possible. Furthermore, professionals do not always have access to the same test environment or the same test equipment. Nevertheless, the main point is that speech intelligibility for the patients in the studies of Der et al. [24], Fan et al. [22], Massi et al. [25] and Sargsyan et al. [14] have improved distinctly with bone conduction devices such as the Bonebridge and BAHA, and with the active middle ear implant, the Vibrant Soundbridge.

The fact that only eight of the original seven hundred and eight publications remained for data extraction shows that hearing treatment is not the focus of research regarding the management of TCS patients. In general, the study cohort size was small or there were only case reports of single individuals, when looking at the included publications [14,20,22,24,25,26,27,28,29]. This is one of the reasons why the results cannot be generalized to TCS patients. In addition, there was unfortunately sometimes no or incomplete information about the hearing solutions that were applied. Therefore, it is difficult to say what is state-of-the-art or what could be achieved with which hearing solutions. Of course, every patient has different preconditions with TCS, and it is important to address the patient’s needs individually. However, it is always good for the patients as well as for the medical practitioners to have sufficient data on which hearing solution was helpful in which specific case to be able to make a more appropriate decision.

A majority of TCS patients experience associated hearing loss [9] and it became clear with the systematic review that this topic is underrepresented or almost not represented in the literature. This is probably due to the variety of other challenges of this pathology. For this reason, it would be desirable to focus more on the management of hearing loss in TCS patients in order to provide them with the best possible living conditions.

## 5. Conclusions

Even though there was little evidence given with only nine remaining publications for the data extraction, it could be shown that active and passive bone conduction devices are the obvious and beneficial choice for people with TCS. Bone conduction devices remain the state of the art for treating conductive hearing loss in TCS patients, according to the summary of the applied hearing solutions. Most frequently utilized were the Bonebridge and the BAHA system in its different modifications. The results of the audiological assessments have shown that the hearing devices applied have provided a clear benefit. Hearing ability and speech comprehension improved in all TCS participants examined with hearing rehabilitation. Nevertheless, the systematic review revealed that there is little to nothing in the literature regarding hearing loss treatment, even though most TCS patients have associated hearing loss. This is most likely a result of the pathology’s several additional difficulties. Therefore, to give TCS patients the optimal living conditions possible, it would be ideal to concentrate more on the research of good management of hearing loss and the acquiring of good speech intelligibility.

## Figures and Tables

**Table 1:** Search terms and outcomes of each search step in the PubMed database.

Search Steps	Search Terms	Hits
1	(((((Treacher Collins syndrome) OR (TCS)) OR (Treacher Collins-Franceschetti syndrome)) OR (mandibulofacial dysostosis)) OR (Franceschetti-Zwahlen-syndrome)) OR (Franceschetti-Klein-syndrome)	5895
2	((((Treacher Collins syndrome) OR (Treacher Collins-Franceschetti syndrome)) OR (mandibulofacial dysostosis)) OR (Franceschetti-Zwahlen-syndrome)) OR (Franceschetti-Klein-syndrome)	2211
3	Limit #2 to humans and English as well as German language articles	2203
4	Filter: to highlight the most recent interventions, a time frame for the last 10 years was chosen from the search date (20 June 2023 at 13:00).	708

**Table 2:** Inclusion and exclusion criteria for identified publications.

Inclusion Criteria	
Population	Subjects of any age, gender, or ethnicity with Treacher Collins syndrome (included are also synonyms for this condition)
Intervention	Hearing loss treatment (any kind of hearing device)
Comparator	Not applicable
Outcomes	Audiological outcomes, language acquisition, hearing ability, general performance, quality of life, satisfaction, subjective outcomes performance (efficacy)
Study design	Randomized or nonrandomized comparative studies, case series, case–control studies, controlled/not controlled before and after studies and interrupted time series analyses Letters, editorials and systematic reviews with no original data, animal, in-vitro and laboratory studies were excluded.
Exclusion Criteria	
	- Different condition (differential diagnosis like Goldenhar syndrome, hemifacial microsomia, Nager syndrome…) - Topic not related to hearing loss or treatment - Publication lacking sufficient information for evaluation - Overlap of data

**Table 4:** Overview of the data collected for the audiological assessments of Treacher Collins patients from the included studies.

Studies	Pure-Tone-Average (PTA)—0.5, 1, 2, 4, kHz (dB)							Speech Recognition							
	Unaided Pre-OP Mean	SD Pre-OP	n	Aided Post-OP Follow-Up (F/U)	Mean	SD Post-OP	n	Assessment	Unaided Pre-OP Mean	SD Pre-OP	n	Aided Post-OP Follow-Up (F/U)	Mean	SD Post-OP	n
Asten et al., 2014 [28]	-	-	-		52.1 dB	20.04 dB	11	Intelligibility—self generated 50 words	-	-	-	-	adults: 98% (93–100), adolescents: 77% (31–99)		adults: 11, adolescents: 8
Der et al., 2018 [24]	66.5 dB	95% CI 64.2–68.9	24	post-OP	31 dB	95% CI 28.2–33.8	24	Soundfield test of speech recognition	29.40%	95% CI 25.2–34.6	24	post-OP	90.70%	95% CI 87.4–93.9	24
				F/U 1 month	25.2 dB	95% CI 23.5–26.9	24					F/U 1 month	96.40%	95% CI 92.7–100	24
Fan et al., 2019 [22]	56.25 dB–60 dB	-	6	F/U 3 months	BAHA: 28.8 dB, Ponto: 36.6. dB, Bonebridge: 27.5 dB	-	6	Mandarin Speech Test Materials (MSTM) at 65 dB SPL	-	-	-	-	BAHA: 44%, Ponto: 51.25%, Bonebridge: 58%		6
Massi et al., 2016 [25]	65 dB (both ears)	-	1	-	30 dB (both ears)	-	-	Soundfield test of speech recognition at 50 dB SPL	48%	-	1	-	-	-	1
Rosa et al., 2016 [29]	60 dB	5.3 dB	9	-	with HA: 26.8 dB	4.1 dB	9	-	-	-	-		-	-	-
				F/U 3 months	with BAHA: 18.3 dB	1.3 dB	4								
Sargsyan et al., 2014 [14]	Right: 66.3 dB, Left: 68.8 dB	-	1	F/U 3 months	Right: 68.8 dB, Left: 66.3 dB	-	1	Mainzer word test, word recognition score (WRS) at 65 dB SPL	0	0	1	-	BAHA softband: 90%	-	1
												F/U 3 months	VSB: 100%	-	1
Shih et al., 2020 [26]	Right: 60 dB, Left: 55 dB	-	1	-	Improved/satisfactory functionality	-	-	-	-	-	-	-	-	-	-
Sikolova et al., 2023 [20]	65 dB (both ears)	-	2	-	30 dB/25 dB	-	2	Speech, Spatial and Qualities of Hearing (SSQ)	4.42/5.97	-	2	F/U 3 months	5.93/7.90	-	2
Zhang et al., 2013 [27]	65 dB–70 dB SPL	-	1	-	20–30 dB SPL	-	1	-	-	-	-	-	-	-	-

## Supplementary Materials

The following supporting information can be downloaded at: https://www.mdpi.com/article/10.3390/jpm14010081/s1, Supplementary file 1, PRISMA Checklist.
